# Cardiac sympathetic denervation in the prevention of genetically mediated life-threatening ventricular arrhythmias

**DOI:** 10.1093/eurheartj/ehac134

**Published:** 2022-03-18

**Authors:** Peter J. Schwartz, Michael J. Ackerman

**Affiliations:** Center for Cardiac Arrhythmias of Genetic Origin and Laboratory of Cardiovascular Genetics, Istituto Auxologico Italiano, IRCCS, Via Pier Lombardo, 22, 20135 Milan, Italy; Department of Cardiovascular Medicine, Division of Heart Rhythm Services (Windland Smith Rice Genetic Heart Rhythm Clinic), Mayo Clinic, Rochester, MN, USA; Department of Pediatric and Adolescent Medicine, Division of Pediatric Cardiology, Mayo Clinic, Rochester, MN, USA; Department of Molecular Pharmacology & Experimental Therapeutics (Windland Smith Rice Sudden Death Genomics Laboratory), Mayo Clinic, Rochester, MN, USA

**Keywords:** Cardiac sympathetic denervation, Catecholaminergic polymorphic ventricular tachycardia, Genetic disorders, Left cardiac sympathetic denervation, Long QT syndrome, Sudden cardiac death

## Abstract

Proper management of patients affected by genetic disorders causing life-threatening arrhythmias is important for several reasons, including even societal ones, given the predominantly young age of those affected. Incorrect management often has dire consequences, ranging from unnecessary psychologic damage for the patients whose life becomes too limited by the fear of sudden death to equally avoidable tragedies when the entire armamentarium of effective therapies is not fully utilized. In this review, we focus primarily on long QT syndrome (LQTS) and catecholaminergic polymorphic ventricular tachycardia (CPVT) and deal specifically with the clinical impact of the most commonly used cardiac sympathetic denervation (CSD), namely left cardiac sympathetic denervation (LCSD). The two of us have used LCSD in the management of our patients with either LQTS or CPVT for a very long time and have been involved in ∼500 such interventions. It is on the basis of this personal and direct experience that we wish to share our views with clinical cardiologists and electrophysiologists, adult and paediatric, and with genetic cardiologists. We will begin by reviewing the history and rationale underlying sympathetic denervation therapy and will continue with a disease-specific intensification of therapy, and then with a discussion on how the impressive efficacy of LCSD should translate into guideline-directed therapy in both current and future guidelines, in order to upgrade the quality of care in the era of precision medicine.

## Graphical Abstract

**Graphical Abstract:**
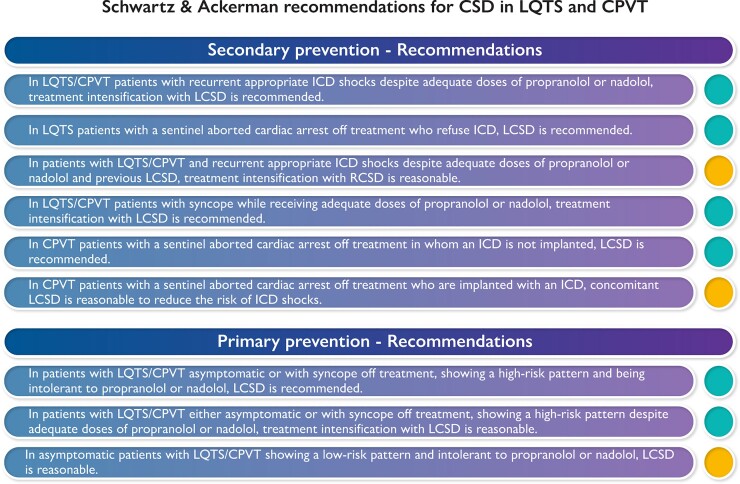
How recommendations for cardiac sympathetic denervation in patients with either long QT syndrome or catecholaminergic polymorphic ventricular tachycardia would look like if they were written by the two of us. LQTS, long QT syndrome; CPVT, catecholaminergic polymorphic ventricular tachycardia.

## Introduction

Cardiac arrhythmias of genetic origin are often deadly.^1^ Their management and prevention are among the potentially most rewarding challenges for paediatric and adult cardiologists and electrophysiologists, and for genetic cardiologists, because—at variance with those representing the inexorable culmination of advanced structural cardiac damage—they do not represent a self-defeating objective.^2^

All too often the therapeutic choice oscillates, dangerously for the patient, between an antiarrhythmic drug (mostly beta-blockers, βBs) and the implantable cardioverter defibrillator (ICD). The quality of life and clinical efficacy seem to be at the two extremes, unable to coexist within one therapeutic approach. However, this is a short-sighted view. Here, we will highlight and discuss a third approach that combines efficacy of treatment and quality of life: namely, cardiac sympathetic denervation (CSD). The two of us have used extensively, and for a long time,^3,4^ left cardiac sympathetic denervation (LCSD) in the management of our patients with life-threatening arrhythmias of genetic origin, primarily long QT syndrome (LQTS), and catecholaminergic polymorphic ventricular tachycardia (CPVT). Thus, we can knowledgeably examine the contribution that LCSD, and occasionally, bilateral CSD, can offer to the management of patients with genetic arrhythmogenic disorders.

We will review the history and rationale underlying sympathetic denervation therapy. We will analyse the data available for LCSD not only for channelopathies, chiefly LQTS and CPVT, but also for other arrhythmogenic conditions as well. We will consider when and how to integrate denervation therapy into disease-specific and genotype-guided intensification of therapy. Finally, we will discuss the impact that the efficacy of this intervention should have on the decisions that clinical cardiologists and electrophysiologists must make when facing a patient who either is not adequately protected by either pharmacological or device therapy, or is not acceptably tolerating those therapies in terms of quality of life.

## Background

### History

The details on the introduction of LCSD in the clinic and of its unforeseen evolution are available.^5–7^ In 1916, Jonnesco performed the first LCSD in a patient with intractable angina pectoris accompanied by cardiac arrhythmias and unexpectedly observed that both the attacks of angina and the arrhythmias disappeared after surgery.^8^ For many years and until the advent of βBs, LCSD remained as an effective therapy for angina. It was only in the 1960s that first Estes and Izlar^9^ and then Zipes *et al.*^10^ successfully used bilateral CSD (stellate ganglion plus seven thoracic ganglia!) in two patients with intractable ventricular tachycardia (VT), but no one followed. The game changer took place in the early 1970s when Moss and McDonald^11^ and then Schwartz and Malliani^12^ started to use LCSD for their patients with LQTS who were refractory to pharmacotherapy.

Despite the clear therapeutic success of these pioneering interventions (both patients remained free of cardiac events for more than 45 years), one of us (P.J.S.) remained the lone standard bearer of LCSD in the setting of genetic arrhythmias. In 2005, the second of us (M.J.A.) joined forces and started to use the thoracoscopic approach to provide LCSD therapy for his LQTS patients.^4^ This approach, far less complex than the retro-pleural approach,^13^ paved the way to minimally invasive surgical cardiac denervation therapy being performed in many different centres. Currently, LCSD is an integral part of the management strategy for both LQTS and CPVT. Meanwhile, in the early 2000s, Shivkumar revived the Estes–Zipes idea and began to use, very successfully, bilateral CSD for intractable VT in patients with structural heart disease such as dilated cardiomyopathies and ischaemic heart disease.^14,15^

### Rationale

As the rationale underlying the clinical use of LCSD has been described in the past,^3,4^ here we will just summarize its main mechanisms of action with the appropriate references for the interested reader. With one exception, all the consequences of LCSD (*Table 1*) derive from the fact that the centrally mediated sympathetic activation can no longer lead to its normal physiologic response, i.e. the release of norepinephrine (NE) upon the ventricular myocardium from the quantitatively dominant left-sided nerves. The localized neural release of NE by the sympathetic terminals, at variance with the rather uniform effect resulting from the blood-borne, adrenal medulla-derived epinephrine, increases the heterogeneity of repolarization and thereby increases the probability of a ventricular arrhythmia by reentry.^16,17^

**Table 1:** Effects of left cardiac sympathetic denervation

Physio- and pathophysiological parameters	Effect
Release of norepinephrine at nerve endings^16,17^	Decrease
Arrhythmias associated with myocardial ischaemia^20^	Decrease
Ventricular refractory period^22^	Increase
Ventricular fibrillation threshold^18^	Increase
Myocardial reactive hyperaemia^21^	Increase
Cardiac performance during exercise^26^	Unaffected

Left cardiac sympathetic denervation increases the ventricular fibrillation (VF) threshold, making it more difficult for a heart to fibrillate.^18^ Probably, this is its single most important effect and it affects much more the onset of VF than a short run of torsades-de-pointes (TdP) VT, the signature arrhythmia of LQTS.^19^ Thus, following LCSD, one can expect a reduction of the occurrence of VF greater than that of otherwise self-terminating arrhythmias (e.g. syncope).

The other effects include a reduction in ischaemia-related arrhythmias^20^ and an increased capability of the coronary bed to dilate:^21^ two factors important especially for patients with ischaemic cardiomyopathy. Other antiarrhythmic effects include the prolongation of ventricular refractoriness,^22^ which also reduces the probability of a reentrant arrhythmia^16,17^ and the reflex increase in cardiac vagal efferent activity^23^ with its well-known antiarrhythmic effect.^24^ On the safety side, it is important to remember that, due to the compensatory effect of right cardiac sympathetic nerves—which is in part reflexly mediated^25^—neither heart rate nor cardiac contractility decrease after LCSD.^26,27^ It is self-evident that the compensation by right cardiac nerves is lost with bilateral CSD. Another clinically relevant point, which often escapes clinicians, is that LCSD represents a pre-ganglionic denervation and, as the synapses are removed and as they do not regenerate, no reinnervation is possible. Similarly, LCSD—at variance with post-ganglionic denervation—is not accompanied by post-denervation supersensitivity,^28,29^ which could have a dangerous proarrhythmic effect.

In contrast to many antiarrhythmic therapies, the precise mechanisms of action of LCSD have been dissected carefully and understood.^6^ This should be reassuring for both doctors and patients.

## Long QT syndrome

Here, we will address the straightforward and the potentially controversial aspects of LCSD in the clinical management of LQTS. To avoid misinterpretations, it seems fair to remind that the two of us have recommended and overseen surgical denervation therapy for nearly 400 patients with LQTS over several decades, a number greater than the total performed worldwide by other investigators.^30,31^ Thus, please understand that after so many years and so many patients, the time has come for unambiguous statements. In this regard, what we are doing could well be regarded as a Consensus Statement of two, or as our own Recommendations (*Graphical Abstract*).

**Figure 1:**
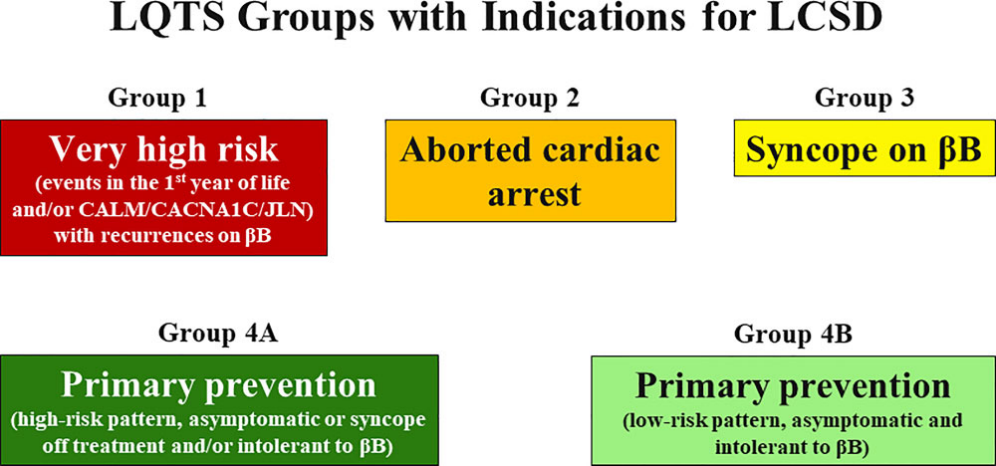
Groups of patients for whom left cardiac sympathetic denervation could be indicated. The level of risk decreases progressively from Group 1 to Group 4B (from red to light green). The rate of success varies within these groups according to Dusi *et al.*^31^ and in some cases an implantable cardioverter defibrillator may become necessary. For Groups 4A and 4B, left cardiac sympathetic denervation is recommended as the primary prevention because these patients are still asymptomatic, but their electrocardiographic pattern suggests a higher or lower risk. See text for details. βB, beta-blocker; CALM, calmodulin; JLN, Jervell and Lange-Nielsen; LCSD, left cardiac sympathetic denervation; LQTS, long QT syndrome.

A preliminary and important point is our recent realization that the probability of success for LCSD varies according to specific subgroups and that it would be naïve to continue to look at the results without considering the clinical/genetic features of the individual patients.^31,32^*Figure 1* outlines the five different scenarios for which LCSD could be considered. Group 1 includes patients regarded as at very high risk because of severe genotypes (e.g. calmodulin-mediated LQTS/CVPT) with recurrences while on the preferred βBs nadolol or propranolol, who require both an ICD and LCSD, the ICD as a safety net and LCSD to decrease as much as possible the appropriate shocks. Group 2 includes patients with an aborted cardiac arrest (ACA) either off or on treatment; in the first case we recommend βBs + ICD, in the second ICD + LCSD as they are already on βBs. Group 3, probably the most important in terms of numbers, are patients who have a syncopal episode while taking βBs; for them, we recommend LCSD with several potential developments, as outlined in Dusi *et al.*^31^ Group 4A refers to patients regarded as at increased risk even though they are either still asymptomatic or have had syncope off therapy and do not tolerate βBs; in this case, we consider a primary prevention LCSD. Group 4B includes asymptomatic patients who appear to be at relatively low risk and are either intolerant to βBs or have expressed clear preference for a one-time surgery instead of a life-long therapy with βBs, after having been duly informed on the potentially different degree of protection.

Updated management of LQTS mandates that cardiologists understand the gene-specific risk and the genotype/phenotype features that define more severe disease requiring treatment intensification,^33,34^ including gene-specific therapy.^35,36^ This reflects the growing role of precision medicine.^37^

Having said that, as a general approach, we will now discuss selected aspects related to LCSD.

### Extent of denervation

**Figure 2:**
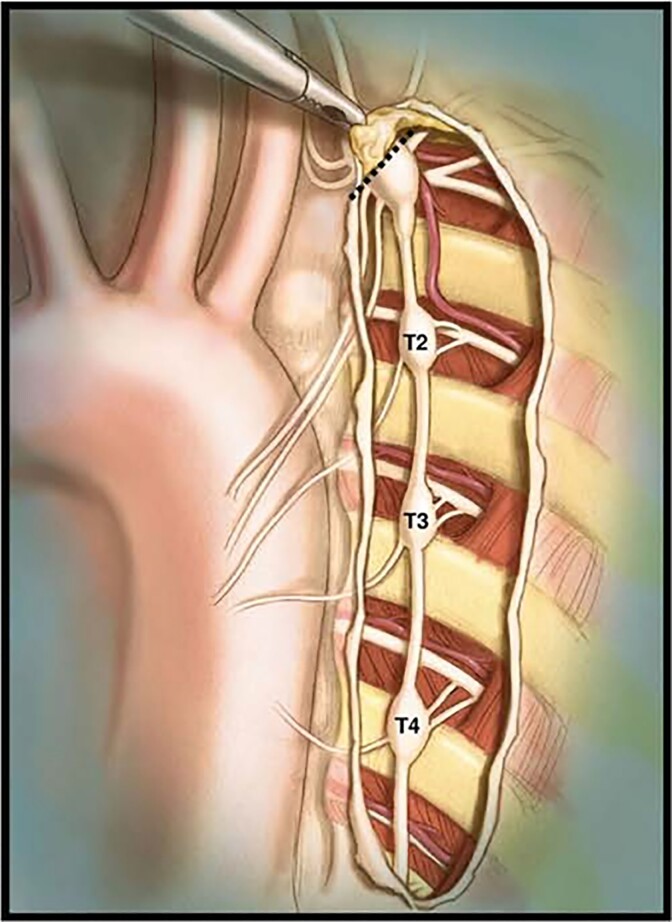
An anatomical drawing of the left cardiac sympathetic chain after exposure through the pleura that is resected during video-assisted thoracic left cardiac sympathetic denervation. The stellate ganglion is located under the superior edge of the incision. The dashed line indicates the resection of the lower half of the left stellate ganglion occurring just above the major lower branches, to minimize the risk of the Horner syndrome. The lower section should take place below T4. Prior to performing the section, lidocaine should be applied on the sympathetic chain. (From Collura *et al.*^4^ with permission.)

The logical extent of surgery is dictated by anatomy and physiology, and it must include the lower half of the stellate ganglion (i.e. T1) together with the thoracic ganglia from T2 to and including T4 (*Figure 2*). The same approach is used at UCLA by Shivkumar’s group, the only other one in the world having a reasonably large experience with CSD.^38^ In our previous reports, with respect to either LQTS or CPVT, incomplete denervation was associated regularly with a higher degree of failures.^39–42^ Thereby, we regard as medically unacceptable and ethically disquieting the recent attempts to ‘simplify’ surgery by either leaving behind both stellate ganglia altogether or T4.^43–45^ In other words, T2–T3 or T3–T4/T2–T5 resection does not constitute LCSD and should be considered essentially ‘sham’ surgery. Ethical Committees worldwide should not authorise ‘experimental surgery’ in humans when the evidence for the correct and effective procedure is well established.

### Clinical efficacy

**Figure 3:**
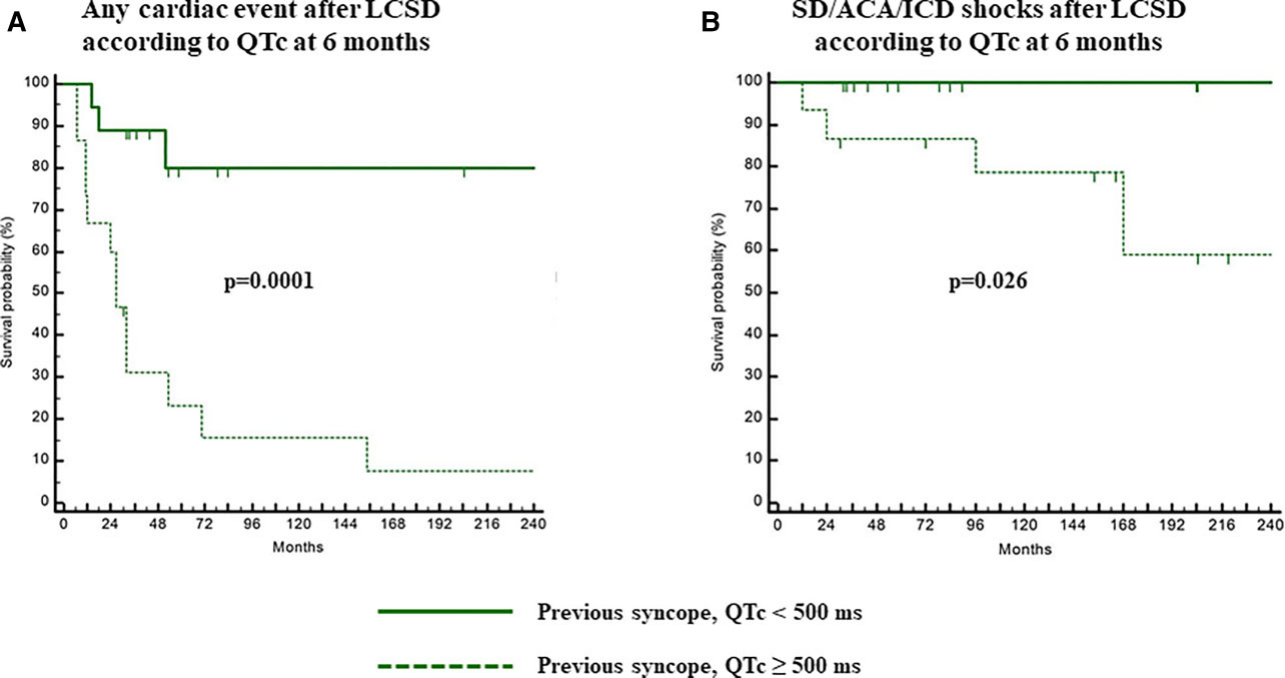
On treatment Kaplan–Meier curves of cumulative survival to any cardiac event (*A*) and to a sudden death/aborted cardiac arrest/implantable cardioverter defibrillator shocks (*B*) after left cardiac sympathetic denervation in LQTS patients with previous syncope/implantable cardioverter defibrillator shocks according to post-left cardiac sympathetic denervation QTc <500 or ≥500 ms. The patients who at 6 months post-left cardiac sympathetic denervation have a QTc <500 ms are at a significantly lower risk for all cardiac events and especially for sudden death/aborted cardiac arrest/implantable cardioverter defibrillator shocks. (Modified from Dusi *et al.*^31^ with permission.) SD, sudden death; ACA, aborted cardiac arrest; ICD, implantable cardioverter defibrillator; LCSD, left cardiac sympathetic denervation.

The efficacy of LCSD is excellent, as shown by our reports,^30,31,39,40,46^ but, like most therapies, does not provide 100% protection. As mentioned above, the clinical presentation offers insights on the probability of success^31^ and on the possible need for adjunct measures such as ICD, atrial pacing, initiating other medications like mexiletine, or proceeding to right-sided cardiac sympathetic denervation (RCSD). Especially important is the predictive role of QTc following LCSD. The most recent data^31^ indicate that whether or not after LCSD, the QTc remains above or below 500 ms makes a difference (*Figure 3*) and that up to half of the patients with a pre-LCSD QTc >500 ms will shorten it by a mean of 60 ms.

Clinical cardiologists should also consider that a consequence of the anti-fibrillatory effect of LCSD is that its efficacy is greater in preventing the deterioration from TdP to VF (which causes cardiac arrest or death) than the onset of a transient episode of TdP (which leads just to syncope). This matters when one has to decide what to do for a patient with syncope on βB therapy.

### Bilateral cardiac sympathetic denervation

The addition of RCSD following LCSD, thus leading to a complete bilateral CSD, can be a useful treatment, when LCSD appears insufficient. We have used it since the mid-1980s in a few cases but with overall rewarding albeit anecdotal results.^31,39,40^ Anecdotal because we have seldom needed to proceed to the RCSD to complete a bilateral CSD in patients with either LQTS or CVPT. In fact, in our joint experience, of the >450 denervations performed to date for either LQTS or CVPT, <20 RCSD followed LCSD. At Mayo Clinic, the approach is to do a re-do LCSD if the surgery was done elsewhere for the patient with a post-LCSD breakthrough cardiac event rather than go straight to the right side.^42^ In doing so, we have found either an untouched left stellate ganglion, a bifid stellate, or the distal T3–T4 sympathetic chain. Accordingly, it is important to avoid misunderstandings: the potential additional value of RCSD does not legitimize at all the performance of a bilateral CSD at outset,^44,45^ without having first assessed whether LCSD is sufficient. There should be caution before depriving, without proven necessity, the patients of the contribution of right-sided cardiac nerves to heart rate control (where they dominate)^47^ and to ventricular contractility.^26^

Right-sided cardiac sympathetic denervation is indicated as an intermediate step before considering ICD implant when LCSD does not provide sufficient protection, and when the patient continues to receive appropriate ICD shocks following LCSD. When, despite LCSD, patients with premature ventricular beats continue to ‘feel them’ and develop anxiety leading to TdP, the performance of RCSD interrupts this traumatizing feedback loop, thanks to the interruption of the cardiac sympathetic afferents which are activated by the ventricular mechanoreceptors.^48^

### Left cardiac sympathetic denervation monotherapy

Already in the 2004 worldwide report on LCSD^40^ in 147 patients, 17 (12%) were treated with LCSD as monotherapy and 82% of them became completely asymptomatic and had a mean QTc shortening of 75 ms. It was since the early days that we knew that some patients could not be treated with βBs, mostly because of severe asthma. The proportion of LQTS patients treated with LCSD monotherapy has significantly increased, nowadays mostly due to intolerance to βBs, 31%^30^ at Mayo Clinic, and 10% in Milan.^31^ Understandably, the majority of these patients (75%) was asymptomatic. Dusi *et al*.^31^ reported LCSD monotherapy because of βBs intolerance in 12 (10%) patients but, interestingly, eight had previous syncope or previous ACA and over a meaningful follow-up of 18 ± 12 years, there were only two patients with syncopal episodes (both with previous ACA).

On the basis of these data, it is now possible to make informed statements about LCSD monotherapy. The ideal therapy for LQTS should always include βBs. However, it is our opinion that in the presence of clear contraindications or true intolerance to βBs, there is now sufficient evidence to allow the patients, including symptomatic ones, to continue with LCSD monotherapy. As the level of protection could be related to the degree of QTc shortening, we advise a stricter follow-up for these patients.

## Catecholaminergic polymorphic ventricular tachycardia

The main problem in the management of CPVT is that it is perhaps the only cardiac disease in which the ICD itself may contribute to not only morbidity but also the patient’s very own mortality.^1,49–52^ Indeed, the pain and fear triggered by ICD shocks, appropriate and inappropriate alike, can precipitate a severe and ultimately fatal electrical storm.^49–52^ The most recent analysis of the effect of ICD implants in patients with CPVT has concluded that ICD use should be limited as much as possible, favouring LCSD.^53^

Left cardiac sympathetic denervation was used for the first time in CPVT patients in 2008^54^ and then an extensive single institution experience from Mayo Clinic was reported in 2012.^55^ This was followed by a multicentre study in 63 patients in 2015^41^ and by several other cases.^51^ The proportion of patients with major cardiac events despite optimal medical therapy was reduced from 100 to 32% after LCSD, and among the 29 patients with a pre-denervation ICD, the rate of shocks dropped by 93% from 3.6 to 0.6 shocks per person per year (*P* < 0.001).^41^ Left cardiac sympathetic denervation is an effective anti-fibrillatory intervention for patients with CPVT. The conclusion of that study was that whenever syncope occurs despite optimal medical therapy, LCSD could be considered the next step rather than an ICD and could complement ICDs in patients with recurrent shocks. This view was fully endorsed by another large multicentre study^51^ and can now be regarded as the expert opinion.

Whenever βBs appear as not adequately protective in CPVT patients, there should be no hesitation whatsoever in proceeding with LCSD. This is a problematic population in which an arrhythmia breakthrough can happen with just a single missed dose of medication, and for which LCSD provides another layer of protection should this occur. For CPVT patients with a sentinel event of sudden cardiac arrest prior to diagnosis, we endorse ‘triple therapy’ with nadolol, flecainide, and LCSD (Mayo Clinic) or nadolol, LCSD, and an ICD (Milan) as reasonable treatment strategies.

## A randomized clinical trial for long QT syndrome or catecholaminergic polymorphic ventricular tachycardia

From time to time someone questions what we call ‘optimal medical treatment’ for LQTS and CPVT, as described above, saying that ‘there has never been a randomized clinical trial (RCT). Yes, there has never been one and hopefully never will be, given that it would be neither ethical nor feasible. As the current treatment options for both diseases are most effective and as for the most severe cases the ICDs represent an effective addition and way out, it would not be ethically acceptable to randomize some patients to a treatment of unproven efficacy. And not even the use of ICDs as a ‘safety net’ would be justifiable, when they are not absolutely necessary, because once implanted the risk of shocks should always be minimized. Finally, it is difficult to imagine how parents would accept to have their child affected by LQTS or CPVT randomized to a treatment of uncertain efficacy which could result in either sudden death or unnecessary ICD shocks. There were no RCTs for penicillin.

## Other monogenic genetic disorders besides long QT syndrome and catecholaminergic polymorphic ventricular tachycardia

It is premature to extend the confidence in LCSD’s therapeutic efficacy beyond LQTS and CPVT at this time. Although we and others have reported LCSD in patients with hypertrophic cardiomyopathy (HCM), arrhythmogenic right ventricular cardiomyopathy,^56^ and even a patient with a classic congenital heart malformation of d-transposition of the great arteries and late onset VT,^55,57,58^ the potential utilization of LCSD in these conditions requires a clear elucidation that the arrhythmic event was triggered by sympathetic activation. If so, and if these disease-associated arrhythmias persisted while already on guideline-directed therapies (GDTs), then LCSD can be considered. At this time, evidence for adequate protection by CSD for HCM and ACM is still insufficient.

## Post-left cardiac sympathetic denervation sequelae and complications

Post-LCSD, patients will experience a drier and warmer left hand as the consequence of the interruption of the sympathetic fibres innervating sweat glands. Rarely, patients will develop a transient harlequin appearance of the face after an aerobic workout or emotional excitement. Concerns about a full Horner syndrome have been exaggerated substantially. A permanent minor left ptosis, that approximates the eyelid asymmetry seen in about 10% of all humans, occurs in about 3–5% of patients. In most patients, a modest ptosis can be seen within the first days after LCSD that gradually resolves over the ensuing months. A major ptosis, requiring an aesthetic surgical correction, occurs in no more than 1% of patients.^13^

The most disturbing consequence of LCSD is neuropathic pain, which was practically absent with the previously used retro-pleural approach^13^ and is probably due to the fact that with the thoracoscopic approach, it is more likely that the surgeon will ‘pull’ the sympathetic chain before cutting it. Approximately 30% of our patients have some level of post-LCSD neuropathic pain which is transient and spontaneously resolves within the first few months after surgery. The administration of low-dose gabapentin starting 24 h before surgery may be a reasonable consideration especially in the phenotypic subset at greatest risk for this side effect (females, age 20–40 years, with prior pain sensitization conditions).

## Quality of life

Even though the primary reason to perform LCSD is to reduce the risk for life-threatening arrhythmias, the physicians’ choice of treatment should always consider the impact on the quality of life^59^ of their patients, especially when they are young. Antiel *et al*.^60^ assessed in 100 patients with LQTS and CPVT whether LCSD had an impact on their quality of life. The vast majority (92%) of patients and families alike were satisfied with their surgery and would recommend it to other patients.^60^

Left cardiac sympathetic denervation improves the quality of life especially among LQTS and CPVT patients with an ICD.^61^ Among 233 LQTS patients with a transvenous ICD, 25% had major adverse events within 5 years after implant.^62^ The effect of LCSD on the number of ICD shocks is impressive, as quantitatively reported in two studies. In five patients the mean yearly rate of shocks per patient dropped post-LCSD by 95% from 29.3 to 3.3 shocks (*P* = 0.02)^40^ and in seven patients it was reduced by >97% from 17 to 0.5 during a mean follow-up of 6 years,^31^ thereby providing some a meaningful therapeutic defence against to the profound post-traumatic stress disorder that can come from the ICD shocks. It had been hoped that the use of a subcutaneous ICD for LQTS and CPVT would have avoided catheter/endovascular-related complications. However, several reasons discourage its use: the potential need for pacing, the inability to prolong detection times, and the probability of more painful shocks.

Although there are a few occasions where we fully support consideration for a prophylactic ICD in asymptomatic patients, before recommending an ICD on the basis of the clinical presentation, it is critical to assess and reassess their risk of a sentinel event once anti-fibrillatory therapies (Bβs + LCSD) and QT shortening (mexiletine) are put in place.

## Left cardiac sympathetic denervation, guidelines, and guideline-directed therapy

Historically, guidelines have not endorsed denervation therapy in general or LCSD in particular with greater than a Class II recommendation. Further, these cardiac society-based, expert opinion-derived guidelines and consensus statements relegated the LCSD as a treatment consideration AFTER the ICD. However, this appropriately changed with the most recent 2017 guidelines from the American Heart Association (AHA), American College of Cardiology (ACC), and Heart Rhythm Society (HRS).^63^ After analysing the extensive, published reports with LCSD in LQTS and CVPT, AHA/ACC/HRS GDT now includes a Class I recommendation for the LCSD as (i) a therapeutic modality for treatment intensification for the patient who has had a disease-associated breakthrough cardiac event including either symptoms while on pharmacotherapy or VF-terminating shocks among those with an ICD and (ii) a *bona fide* treatment alternative in those who do not tolerate βB therapy or have contraindications to βB therapy.

Importantly, the AHA/ACC/HRS guidelines struck equipoise in each situation as it endorsed that the clinician could consider modification of drug therapy, LCSD, or device therapy with an ICD as equally reasonable options for treatment intensification rather than directing/mandating that the ICD is second in line and that the LCSD can only be considered *AFTER* the ICD has been installed. This enlightened approach is counteracting the rapid reflex towards an ICD disquietingly seen in North America and Europe.^62,64^ Along the same lines, but even more forcefully, is the 2021 PACES Expert Consensus statement just published.^65^ Whether or not the upcoming European guidelines will strike a similar level of equipoise for the LCSD in GDT for both LQTS and CPVT remains to be determined.

A very recent study^66^ on 3035 patients enrolled in the US portion of the International LQTS Registry^67^ reported that those with ICDs had a lower risk of death and concluded by supporting ICD implantation in LQTS patients with ACA and with syncope on βBs, and suggesting the same even for those with syncope off βBs. Data from registries have significant limitations because, while reflecting a ‘real-world’ scenario, with a non-uniform management strategies the outcomes are at great variance with those observed in highly experienced centres managing daily patients with LQTS on fully personalized, optimized GDTs.^68^ It is our view that most patients with LQTS and CPVT do NOT need and should NOT receive an ICD.

It is fair to remember that the Task Force of the European Society of Cardiology on the legal implications of medical guidelines^69^ has stated that those who generate them should be respected for their expertise which, for the indications in favour or against CSD, should be highly specific.

Meanwhile, we share what would constitute ‘recommendations’ related to the potential use of CSD in the management of either LQTS or CPVT (*Graphical Abstract*) if it were up to us to decide.

## Right to be informed

For the LQTS and CPVT patients not fully protected by βBs, the availability of both LCSD and ICDs makes it imperative that patients and families know about LCSD and the additional protection it can afford with fewer adverse events compared with ICD. The right of the patients is matched by the responsibility of the physicians to provide adequate and fair information, at risk of medico-legal consequences.^70^

## Conclusions

Our conclusions are the logical consequence of having witnessed, presented, and discussed the impressive clinical impact of LCSD for different subgroups of patients affected by LQTS or CPVT. Our views of what should be done are expressed in what we have unabashedly defined as ‘our own recommendations’, which should be viewed not so much as a criticism of what has been done so far but as a constructive proposal to make future guidelines more representative of what our experience has taught us and should teach others.

Finally, we cannot hide our dismay to note how so many cardiologists around the world, even from some of the most advanced countries, are ready to implant ICDs in a large number of LQTS/CPVT patients but seem unable to offer them the alternative of LCSD. Just because an ICD can be implanted almost anywhere is not a compelling justification to keep families from being fully informed of the GDTs available to them.
